# Effect of pooled tracheal sample testing on the probability of *Mycoplasma hyopneumoniae* detection

**DOI:** 10.1038/s41598-024-60377-z

**Published:** 2024-05-03

**Authors:** Ana Paula Serafini Poeta Silva, Robert Mugabi, Marisa L. Rotolo, Seth Krantz, Dapeng Hu, Rebecca Robbins, Deanne Hemker, Andres Diaz, A. W. Tucker, Rodger Main, Jean Paul Cano, Perry Harms, Chong Wang, Maria Jose Clavijo

**Affiliations:** 1 Veterinary Diagnostic and Population Animal Medicine, Iowa State University, Ames, IA USA; 2 PIC®, Hendersonville, TN USA; 3 Tosh Farms, Paris, TN USA; 4 College of Liberal Arts and Sciences, Iowa State University, Ames, IA USA; 5 University of Cambridge, Cambridge, UK; 6 Pipestone Systems, Pipestone, MN USA

**Keywords:** Surveillance, *Mycoplasma hyopneumoniae*, Pooled sample, Probability of detection, PCR, Infectious diseases, Epidemiology

## Abstract

Tracheal pooling for *Mycoplasma hyopneumoniae* (*M. hyopneumoniae*) DNA detection allows for decreased diagnostic cost, one of the main constraints in surveillance programs. The objectives of this study were to estimate the sensitivity of pooled-sample testing for the detection of *M. hyopneumoniae* in tracheal samples and to develop probability of *M. hyopneumoniae* detection estimates for tracheal samples pooled by 3, 5, and 10. A total of 48 *M. hyopneumoniae* PCR-positive field samples were pooled 3-, 5-, and 10-times using field *M. hyopneumoniae* DNA-negative samples and tested in triplicate. The sensitivity was estimated at 0.96 (95% credible interval [Cred. Int.]: 0.93, 0.98) for pools of 3, 0.95 (95% Cred. Int: 0.92, 0.98) for pools of 5, and 0.93 (95% Cred. Int.: 0.89, 0.96) for pools of 10. All pool sizes resulted in PCR-positive if the individual tracheal sample Ct value was < 33. Additionally, there was no significant decrease in the probability of detecting at least one *M. hyopneumoniae*-infected pig given any pool size (3, 5, or 10) of tracheal swabs. Furthermore, this manuscript applies the probability of detection estimates to various real-life diagnostic testing scenarios. Combining increased total animals sampled with pooling can be a cost-effective tool to maximize the performance of *M. hyopneumoniae* surveillance programs.

## Introduction

*Mycoplasma hyopneumoniae* (*M. hyopneumoniae*) is the etiological agent of porcine enzootic pneumonia and an important pathogen in the porcine respiratory disease complex^1^. *M. hyopneumoniae* is recognized as one of the most impactful bacterial agents in the United States of America (USA)^2^. The Swine Disease Reporting System, a project that aggregates diagnostic data from six veterinary diagnostic laboratories in the USA, reported a *M. hyopneumoniae* detection rate of 9.17% in sow farms and 12.27% in wean-to-market pigs in January 2024^3–5^.

*M. hyopneumoniae* is a challenge for pig production because it decreases growth performance, especially in the presence of co-infections, increases antibiotic use, and is one of the main drivers of wean-to-finish mortality^6–9^. As an example, in *M. hyopneumoniae*-infected herds in the USA, a pig producer may see a decrease of $0.63–$10.12 per market pig, compared to *M. hyopneumoniae*-free herds^9^. Boeters et al.^10^ showed that the cost of *M. hyopneumoniae* ranged between €2–€8 per growing pig in Europe. Magalhães et al.^7^ revealed a 4% higher wean-to-finish mortality in herds infected with *M. hyopneumoniae* compared to *M. hyopneumoniae*-negative herds in one swine system in the USA. Additionally, Magalhães et al.^11^ demonstrated that *M. hyopneumoniae* status in weaned pigs was a predictor for nursery mortality using machine learning models. Thus, control and elimination of this agent from pig populations is essential for the sustainability of the industry.

Managing *M. hyopneumoniae* infection commonly involves establishing sow herd immunity through strategic measures such as gilt acclimation (intentional infection of gilts at an early age), targeted medication, and vaccination of both sows and pigs^12,13^. *M. hyopneumoniae* vaccines accessible in the market are widely used globally and are composed of inactivated or adjuvanted whole-cell bacterins that confer only partial protection^12^. Consequently, the challenge in controlling and eliminating *M. hyopneumoniae* arises due to the lack of vaccines capable of generating sterilizing immunity and limiting the prolonged infection and shedding of *M. hyopneumoniae*^14^. Further, Dos Santos et al.^15^ and Betlach et al.^16^ showed a high genetic variability among *M. hyopneumoniae* strains circulating in the USA commercial herds, which can contribute to varying degrees of vaccine efficacy^17^.

Given the constrains on effectively controlling *M. hyopneumoniae* and the significant cost associated with the disease, producers target elimination from farms or even eradication from the whole production system^18–20^. Further supporting the elimination of this pathogen, Silva et al.^21^ demonstrated that eliminating *M. hyopneumoniae* from pig populations was economically advantageous and provided investment return in less than eight months. Thus, across the USA swine industry, producers are working towards *M. hyopneumoniae*-negative or -controlled statuses for their herds^22^. In both scenarios, it is critical to implement a sensitive and cost-effective surveillance program, e.g., through repetitive collection and diagnostic testing of antemortem specimens^23^.

Due to the slow spread in the pig population and accompanying delay in clinical signs, confidence in detecting this pathogen through surveillance is paramount to maintaining health status^23–25^. While the gold standard diagnostic test for bacterial agents is isolation from infected tissues or specimens, the fastidious nature of *M. hyopneumoniae* implies a low level of success^26,27^. Bacterial culture of *M. hyopneumoniae* can take 4–8 weeks to achieve a reasonable amount of growth and success is variable as *M. hyopneumoniae* can easily be overgrown by other bacteria^26^. Thus, the two most frequently employed diagnostic methods for *M. hyopneumoniae* surveillance are detection of antibodies using an enzyme-linked immunosorbent assay (ELISA) and nucleic acid detection by polymerase chain reaction (PCR)^23,27,28^.

Historically, monthly serum samples tested with ELISA have been implemented in swine herds to monitor health status^22,23,27^. This approach, while economical, can result in delayed detection due to the length of time needed for antibody production, as detection of serum antibodies can vary between 3 and 8 weeks, and unexpected results due to cross-reactions with other mycoplasmas, e.g., *M. flocculare* can result in false positive reactors^29,30^. Additionally, the lack of Differentiating Infected from Vaccinated Animals (DIVA) vaccines for *M. hyopneumoniae* can preclude the use of ELISA testing in vaccinated populations.

Research has shown that tracheal samples tested by PCR allow for detection of the agent quicker and provides a higher diagnostic sensitivity than ELISA testing of serum samples^23,27,31,32^. Tracheal samples are recognized as the most effective approach when the sampling objective is to maximize diagnostic sensitivity and specificity^23^. A single PCR test is almost six times more expensive than a single commercial serum ELISA test, as reported elsewhere^23^.

A common practice within PCR-based testing is to reduce the cost of testing by pooling multiple samples. Pooling samples is defined as a process where a portion of samples from individuals are combined and tested as one sample or pool. One pool may consist of a minimum of2 to an unlimited number of individual samples, depending on the sampling objective^33^. If the pool is negative, then all individuals are considered negative^33^. Conversely, if the pool is positive, the clinician can decide to test all samples individually to identify which samples within the pool are positive^33^. First developed for surveillance of syphilis during World War II^34^, this approach has been applied effectively in veterinary population medicine for surveillance of various pathogens, including porcine reproductive and respiratory syndrome virus (PRRSV) in pigs, bovine viral diarrhea virus (BVDV) in cattle, and influenza A virus (IAV) in broiler chickens^35–38^.

In addition to the decrease in cost and increase in testing efficiency and positive predictive value, pooled testing can be quite effective for low disease prevalence scenarios^39,40^. Given the slow transmission rate of *M. hyopneumoniae,* large sample sizes are needed for timely detection of the agent^23–25,41^. According to Clavijo et al.^23^, after 28 days post-exposure in a naïve population, 30 tracheal samples ($750, assuming $30.00 per PCR test in USA dollar) provided ~ 52% probability of detecting *M. hyopneumoniae* DNA versus 120 tracheal samples ($3000), which provided ~ 91% probability^8^. As demonstrated previously, testing large sample sizes may be cost prohibitive and supports implementing a pooling strategy, however the effect of test performance is unknown. Pooling can reduce the analytical sensitivity depending on the disease prevalence, individual sample pathogen load, and pool size due to dilution^35,38,42^.

Sponheim et al.^42^ demonstrated that tracheal pools of 5 improve *M. hyopneumoniae* DNA detection at later stages of natural infection (> 113 days post-infection). This promising result generated further opportunities of increasing pool size or evaluating the pooling effect at low (< 1%) prevalence scenarios. Therefore, the objective of this study was to evaluate the effect of various pooling sizes (3 vs 5 vs 10) of tracheal samples on *M. hyopneumoniae* DNA detection and to develop probability of *M. hyopneumoniae* detection estimates for tracheal samples pooled by 3, 5 and 10.

## Materials and methods

### Study design

**Figure 1:**
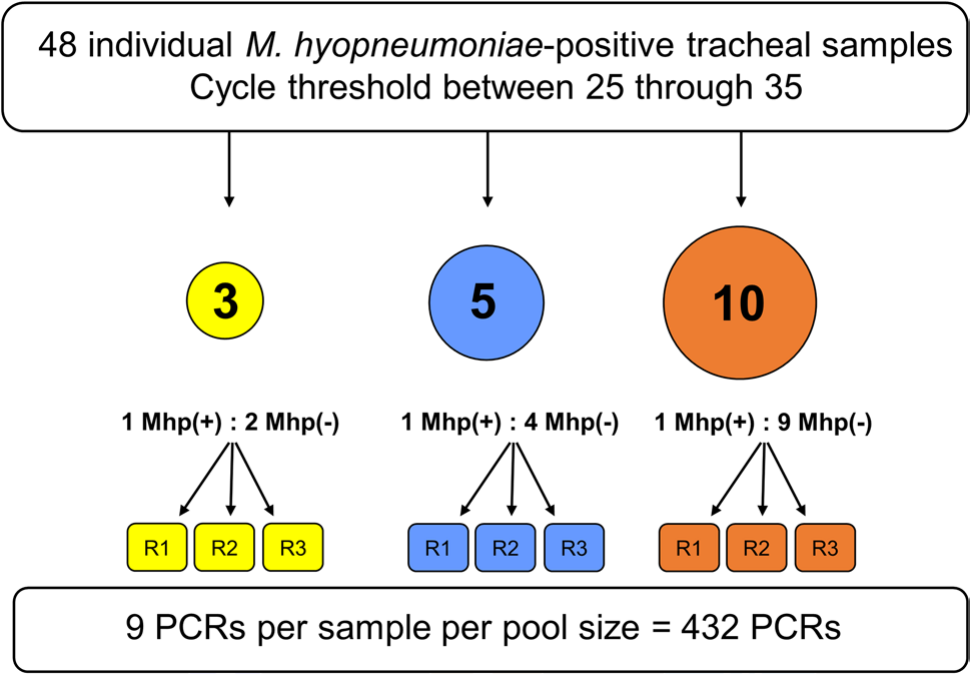
Study design of tracheal pooling for *Mycoplasma hyopneumoniae* DNA detection by polymerase chain reaction (PCR). Individual *Mycoplasma hyopneumoniae*-positive tracheal samples ([Mhp(+)]; [*n* = 48]) were pooled by of 3, 5, and 10 times in *Mycoplasma hyopneumoniae*-negative tracheal samples (Mhp(−)) and tested in triplicate (R1, R2, and R3 represent the replicates 1, 2, and 3, respectively).

Tracheal samples derived from a wean-to-finish population of known positive *M. hyopneumoniae* status (based on observed clinical signs and historical PCR-positive testing) were tested by PCR. A total of 50 tracheal samples with PCR cycle threshold (Ct) values between 27 and 36 were pre-selected and re-tested individually. After the individual re-test, two out of 50 expected positive samples tested negative for *M. hyopneumoniae* DNA and then were excluded from the pooling evaluation. Thus, 48 *M. hyopneumoniae* PCR-positive tracheal samples with varied Ct values (25 < Ct < 35) were used in the pooling evaluation. Three pool sizes were evaluated for each Ct unit, pools of 3, 5 and 10 (Fig. 1). To mimic field conditions and control for sample diversity, 70 tracheal samples collected from an *M. hyopneumoniae* confirmed negative population were combined and homogenized to represent the negative samples in the pools, along with the positive samples. All pool sizes were tested in triplicate. Diagnostic sensitivities of pooled tracheal samples were estimated using a Bayesian binomial model. The effect of pool size and initial Ct value of the individual sample on Ct values from pooled samples were compared using mixed linear regression. Finally, the effect of pooling was evaluated in terms of probability of detecting *M. hyopneumoniae* infected pigs using simulated field data from a wean-to-finish site using a hierarchical latent spatial piecewise exponential model^23^.

### Sample collection, processing, and pooling

Tracheal samples were collected by restraining the pig with a snare and mouth speculum, and introducing a single-use catheter into the trachea, as previously described^25,31,42^. Field tracheal samples (*n* = 50) were initially tested using a *M. hyopneumoniae* PCR and then stored at – 80 °C (no treatment was performed prior to freezing). To mimic sample matrix and reduce variation between samples and pool size, *M. hyopneumoniae* PCR-negative samples (*n* = 70) were homogenized in a sterile container and aliquoted into 5 mL sterile culture tubes. The aliquoted negative tracheal sample tubes were then frozen at – 80 °C (no treatment was performed prior to freezing). On the pooling day, a sub-sample of the negative tracheal sample tubes, in addition to the individual positive tracheal samples, were thawed to be used. Following the re-testing of individual samples, a total of 48 *M. hyopneumoniae* PCR-positive tracheal samples (25 < Ct < 35) were pooled by 3, 5, and 10, as follows: to create a pool of size 3, 125 µL of *M. hyopneumoniae*-positive sample was combined in 250 µL *M. hyopneumoniae*-negative sample mixture; 125 µL of *M. hyopneumoniae*-positive sample was combined in 500 µL *M. hyopneumoniae*-negative sample mixture (pool size of 5); and 125 µL of *M. hyopneumoniae*-positive sample was combined in 1125 µL *M. hyopneumoniae*-negative sample mixture (pool size of 10). All pools were run in triplicate, resulting in a total of 432 M*. hyopneumoniae* PCR tests (48 samples × 3 pool sizes × 3 replicates).

### Sample testing

DNA from tracheal samples used in this study (50 individual tracheal samples generated from known *M. hyopneumoniae*-positive samples [pre-selection tests and re-tests], 70 from known *M. hyopneumoniae*-negative samples, and 432 pooled tracheal samples tested in pool sizes of 3, 5, and 10) was extracted using the commercial kit MagMAX™-96 Pathogen RNA/DNA kit (Applied Biosystems, Carlsbad, CA USA) and amplified using the TaqMan® Fast Virus 1-Step Master Mix with the addition of the AmpliTaq® 360DNA Polymerase (5 U/µL) enzyme (Thermo Fisher Scientific, Waltham, MA USA), and primer/probe as previously described for Mhp183^43^. DNA amplification was performed on the automated Applied Biosystems® 7500 Real-Time PCR (ThermoFisher Scientific). A PCR result was considered valid if the internal positive control Ct was < 36, and tracheal samples were considered positive for *M. hyopneumoniae* DNA when Ct was < 37^43^.

### Statistical analyses

Analyses and graphs were performed in R (R program version 4.2.1, R core team 2022) assuming statistical significance when the alpha level was ≤ 0.05 (*p* ≤ 0.05).

### *Mycoplasma hyopneumoniae* PCR pool sensitivity

The sensitivity of the pools was estimated using a Bayesian parameter estimator for a binomial model. Two assumptions were incorporated: (1) one sample within a pool size was considered positive and (2) pooled samples were assumed to be independent within and across pool sizes. A prior distribution for the diagnostic sensitivity was assumed to account for misclassification in PCR results from individual tracheal samples, i.e., Beta ~ (0.965). Diagnostic pool sensitivity of each pool size was defined by the posterior expectation of the binomial probability model, and a 95% credible interval (Cred. Int) was based on the 2.5th and 95th percentiles of the posterior binomial distribution.

### Effect of pool size and individual Ct value on pooled sample Ct value

The effect of pooling on Ct values was compared by using a linear mixed-effect regression model and controlled by the initial Ct value of the individual sample. Ct values of pooled samples were considered the dependent variable, pool size (3, 5, and 10), initial individual Ct values, and their interaction as independent variables, and the sample (3 replicates) was considered a repeated measure (*nlme* R package). Marginal means (least-square means, LS means) estimated from the linear mixed model were compared among pairs using Tukey–Kramer test (*emmeans* R packages). Residual normality and homoscedasticity assumptions were evaluated using Q–Q plot and residuals versus fitted plot, respectively.

### *Mycoplasma hyopneumoniae* probability of detection using pooled samples

The estimated diagnostic sensitivity of pooled tracheal samples by pool size was utilized to model probability of detection of *M. hyopneumoniae* in tracheal samples using simulated field data. The simulated field data was based on a conservative scenario of *M. hyopneumoniae* prevalence. The scenario was one of 1250 growing pigs was *M. hyopneumoniae*-infected, (prevalence of 0.08%). As described elsewhere^23^, the probability of detecting at least one *M. hyopneumoniae-*infected under the condition of a 1250-head growing pig farm was estimated using a hierarchical latent spatial piecewise exponential model^44^. The model simulated (*n* = 10,000 iterations) the spread of *M. hyopneumoniae* infection in a barn given one initially infected pig (using *rjags* R package). In this study, the modeling was modified by applying the 95% credible interval lowest limit from estimated diagnostic pool sensitivity given different sample sizes collected (between 15 through 120 samples), and then pooled by 3, 5, and 10. The tracheal sampling approach was a fixed spatial design, with the number of samples equally distributed across the entire space with one or more randomly selected pigs per pen depending on the sample size of tracheal samples^23,44,45^.

### *Mycoplasma hyopneumoniae* diagnostic testing scenarios

The probability of detection estimates derived from Clavijo et al.^23^ and pooled tracheal samples derived from this study were contrasted across four main *M. hyopneumoniae* testing scenarios; quarantine testing, disease elimination, breeding stock active surveillance for marketing weaned pigs and grow-out pigs. The testing scenarios were described in terms of testing objective, sample type and size, diagnostic costs, and the probability of detection estimates(individual test and three pool sizes of tracheal samples).

## Results

### Diagnostic sensitivity

Two (2/50) individual positive tracheal samples with initially high Ct values (36.6 and 36.8) were *M. hyopneumoniae* PCR-negative after re-testing and were excluded from the analysis. A total of 432 pooled samples were used in the sensitivity estimation. From those, 403 pooled samples were *M. hyopneumoniae* PCR-positive, 137 of 144 (95.1%) positive tracheal samples in pools of 3, 136 of 144 (94.4%) positive tracheal samples in pools of 5, and 130 of 144 (90.3%) positive tracheal samples in pools of 10. The pool sensitivity was estimated at 0.96 (95% Cred. Int. 0.93, 0.98) for pools of 3, 0.95 (95% Cred. Int. 0.92, 0.98) for pools of 5, and 0.93 (95% Cred. Int. 0.89, 0.96) for pools of 10.

### Effect of pool size and individual Ct value on pooled sample Ct value

**Figure 2:**
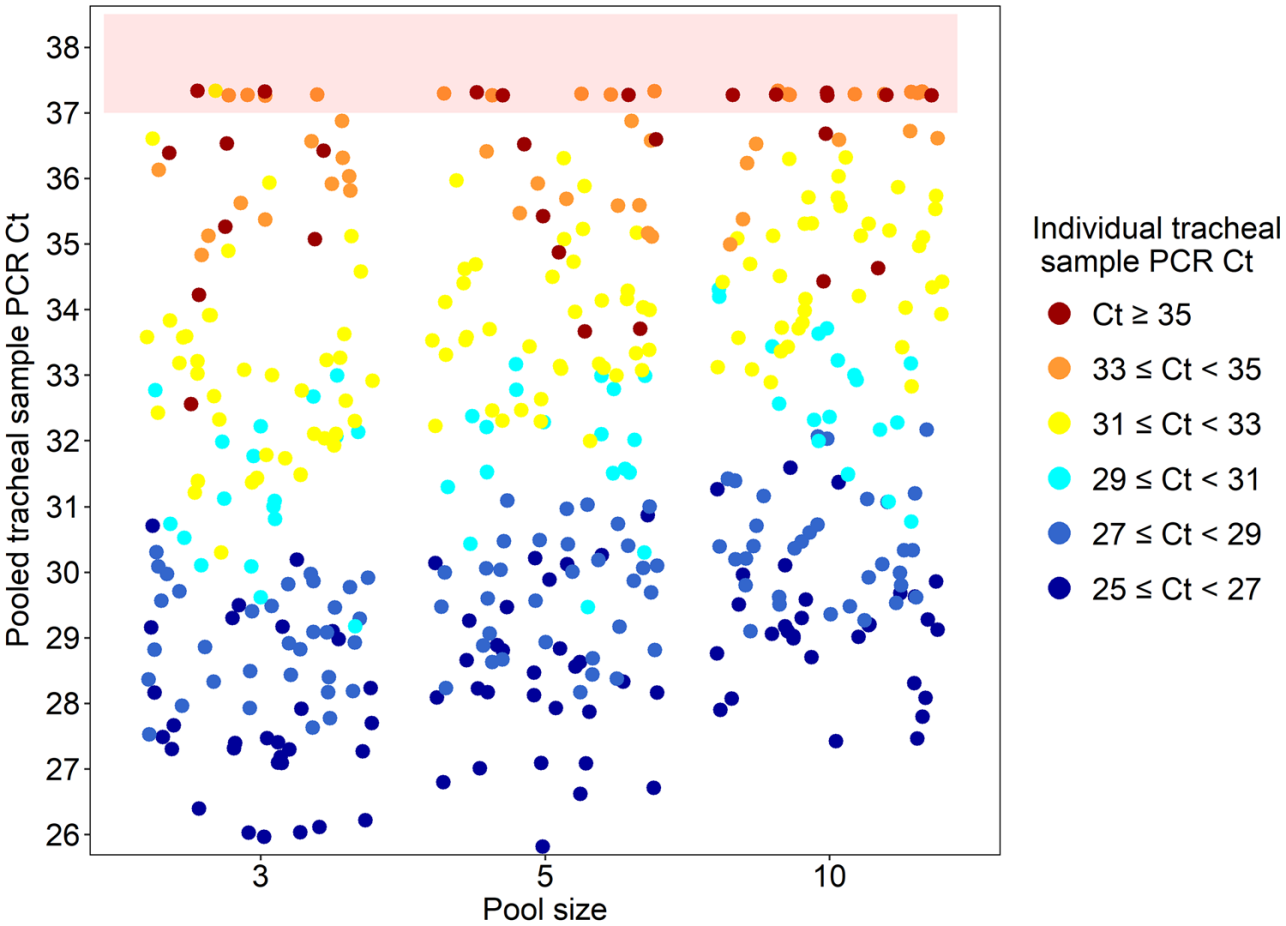
Variation of *Mycoplasma hyopneumoniae* polymerase chain reaction (PCR) cycle threshold (Ct) values in pooled tracheal samples related to initial individual tracheal sample PCR Ct value by pool size. The red shaded area represents the pooled samples that tested PCR-negative for *Mycoplasma hyopneumoniae* (PCR Ct > 37).

Ct values of the 48 PCR-positive individual samples ranged from 25.3 to 35.9. All pool sizes resulted in a positive classification if the individual sample was Ct < 33, in the exception of one sample from pool size 3 (Fig. 2). Regardless of the initial Ct value from the individual sample, Ct value increased as the pool size increased (*p* < 0.0001). Ct value differences of pooled tracheal samples compared to individual sample Ct value by pool size are shown in Table 1. Additionally, the magnitude of Ct loss in pooled samples was numerically higher in lower initial Ct values.

**Table 1:** *Mycoplasma hyopneumoniae* DNA detection and polymerase chain reaction (PCR) cycle threshold (Ct) values in 48 individual and 432 pooled tracheal samples by pool sizes (3, 5, and 10).

Individual Ct value (re-test)	Number of samples per Ct	Pool size	Ct difference from individual sample^1^	Positivity rate by individual Ct (positive/total replicates)
25	4	1	1^a^	
		3 5 10	+ 1.71^b^ + 2.53^c^ + 3.36^d^	12/12 (100%)
				12/12 (100%)
				12/12 (100%)
26	6	1	1^a^	
		3 5 10	+ 1.62^b^ + 2.40^c^ + 3.21^d^	18/18 (100%)
				18/18 (100%)
				18/18 (100%)
27	8	1	1^a^	
		3 5 10	+ 1.54^b^ + 2.27^c^ + 3.06^d^	24/24 (100%)
				24/24 (100%)
				24/24 (100%)
28	3	1	1^a^	
		3 5 10	+ 1.45^b^ + 2.14^c^ + 2.90^d^	9/9 (100%)
				9/9 (100%)
				9/9 (100%)
29	2	1	1^a^	
		3 5 10	+ 1.36^b^ + 2.02^c^ + 2.75^d^	6/6 (100%)
				6/6 (100%)
				6/6 (100%)
30	4	1	1^a^	
		3 5 10	+ 1.28^b^ + 1.88^c^ + 2.60^d^	12/12 (100%)
				12/12 (100%)
				12/12 (100%)
31	8	1	1^a^	
		3 5 10	+ 1.19^b^ + 1.76^c^ + 2.45^d^	24/24 (100%)
				24/24 (100%)
				24/24 (100%)
32	5	1	1^a^	
		3 5 10	+ 1.11^b^ + 1.63^c^ + 2.30^d^	14/15 (93.3%)
				15/15 (100%)
				15/15 (100%)
33	2	1	1^a^	
		3 5 10	+ 1.02^b^ + 1.50^c^ + 2.15^d^	2/6 (33.3%)
				2/6 (33.3%)
				3/6 (50%)
34	3	1	1^a^	
		3 5 10	+ 0.93^b^ + 1.37^c^ + 2.00^d^	7/9 (77.8%)
				6/9 (66.7%)
				4/9 (44.4%)
35	3	1	1^a^	
		3 5 10	+ 0.85^b^ + 1.24^c^ + 1.85^c^	7/9 (77.8%)
				6/9 (66.7%)
				3/9 (33.3%)

^1^Ct difference (least square means) of *M. hyopneumoniae* PCR-positive pooled tracheal samples compared to individual tracheal samples by Ct value unit estimated using linear mixed model. Different letters (in order, a–d) represent statistical difference at *p* ≤ 0.05.

### Probability of detection estimates using pooled tracheal samples

The pool sensitivity used to model probability of detection estimates given sample size and day post-exposure was 0.93 for pools of 3, 0.92 for pools of 5, and 0.89 for pools of 10. The probability of detecting at least one *M. hyopneumoniae* DNA-positive pig in a 1250-head wean-to-finish barn using individual tracheal samples and pooled tracheal samples are given in Table 2. Overall, the probability of detection estimates of pooled tracheal samples decreased between 0.01 and 0.03 compared to the individual probability of detection estimates.

**Table 2:** Barn-level probability of detecting at least one *Mycoplasma hyopneumoniae* DNA positive result based on polymerase chain reaction (PCR) using pooled tracheal samples (3, 5, and 10) given one pig was initially infected by day post-infection and number of samples collected using a fixed spatial approach (at least one tracheal sample per pen).

		Day post-exposure										
		0	7	14	21	28	35	42	49	56	63	70
# collected	# tested	Individual tracheal sample										
15	15	0.01	0.08	0.14	0.22	0.31	0.44	0.54	0.68	0.76	0.87	0.90
30	30	0.03	0.15	0.26	0.41	0.53	0.68	0.76	0.87	0.92	0.96	0.98
60	60	0.05	0.28	0.45	0.63	0.76	0.87	0.92	0.96	0.98	0.99	1.00
90	90	0.07	0.39	0.57	0.77	0.86	0.94	0.97	0.99	0.99	1.00	1.00
120	120	0.09	0.47	0.65	0.84	0.91	0.96	0.99	0.99	1.00	1.00	1.00
# collected	# tested	Pools of 3 tracheal sample										
15	5	0.01	0.08	0.14	0.23	0.31	0.44	0.52	0.66	0.74	0.86	0.90
30	10	0.02	0.15	0.25	0.40	0.51	0.66	0.75	0.86	0.90	0.95	0.97
60	20	0.04	0.28	0.43	0.63	0.74	0.86	0.91	0.96	0.98	0.99	1.00
90	30	0.07	0.37	0.56	0.76	0.85	0.93	0.96	0.99	0.99	1.00	1.00
120	40	0.09	0.46	0.65	0.84	0.90	0.96	0.98	0.99	1.00	1.00	1.00
# collected	# tested	Pools of 5 tracheal sample										
15	3	0.01	0.07	0.13	0.22	0.30	0.42	0.51	0.65	0.73	0.85	0.90
30	6	0.02	0.15	0.25	0.40	0.51	0.65	0.74	0.85	0.90	0.95	0.97
60	12	0.04	0.27	0.43	0.61	0.73	0.86	0.91	0.95	0.97	0.99	0.99
90	18	0.07	0.36	0.55	0.74	0.85	0.93	0.96	0.99	0.99	1.00	1.00
120	24	0.08	0.44	0.63	0.82	0.90	0.96	0.98	0.99	1.00	1.00	1.00
# collected	# tested	Pools of 10 tracheal sample										
15	2	0.01	0.10	0.17	0.28	0.36	0.50	0.59	0.72	0.79	0.87	0.91
30	3	0.02	0.14	0.24	0.39	0.49	0.63	0.72	0.83	0.88	0.95	0.97
60	6	0.04	0.26	0.42	0.61	0.73	0.84	0.90	0.95	0.97	0.99	0.99
90	9	0.07	0.36	0.54	0.74	0.83	0.93	0.96	0.98	0.99	1.00	1.00
120	12	0.08	0.45	0.62	0.81	0.89	0.96	0.98	0.99	1.00	1.00	1.00

Individual probabilities of detection were extracted from Clavijo et al.^23^**.**

### *Mycoplasma hyopneumoniae* diagnostic testing scenarios

Four main diagnostic testing scenarios for *M. hyopneumoniae* in pig populations are described in Table 3. For quarantine testing, a one-time test collection of 60 tracheal samples at 35 days post-placement resulted in 0.87 probability of detection for individually tested tracheal samples with a total cost of $1800. In contrast, with the same testing scenario and sample scheme, using pool size of 10 resulted in 0.84 probability of detection with a testing cost of $180.

**Table 3:** Comparison of *Mycoplasma hyopneumoniae* probability of detection estimates between tracheal sample individual vs. pooled testing, sample size, sample type and test costs across four main *M. hyopneumoniae* diagnostic testing scenarios.

Diagnostic testing scenarios^1^		Testing approach	Sample type	Sample size^2^	Probability of detection^3^				Testing cost (# of tests)^4^			
					Individual^3a^	Pooling^3b^			Individual^4a^	Pooling^4b^		
						3	5	10		3	5	10
Quarantine testing		One-time test	Trachea	120 @ 21d^2a^	0.84	0.84	0.82	0.81	$3600	$1200 (40)	$720 (24)	$360 (12)
				60 @ 35d^2a^	0.87	0.86	0.86	0.84	$1800	$600 (20)	$360 (12)	$180 (6)
				30 @ 49d^2a^	0.87	0.86	0.85	0.83	$900	$300 (10)	$180 (6)	$90 (3)
			Serum	120 @ 35d^2a^	0.60	NA			$660	NA		
				120 @ 42d^2a^	0.79				$495			
				120 @ 49d^2a^	0.88				$330			
Breeding stock active surveillance	Marketing weaned pigs	Monthly (source farm)	Trachea	30^2b^	0.41	0.40	0.40	0.39	$900	$300 (10)	$180 (6)	$90 (3)
				60^2b^	0.63	0.63	0.61	0.61	$1800	$600 (20)	$360 (12)	$180 (6)
	Grow-out pigs	On-time test prior to the movement	Trachea	30^2c^	0.76	0.75	0.74	0.72	$330	$300 (10)	$180 (6)	$90 (3)
				60^2c^	0.92	0.91	0.91	0.90	$1800	$600 (20)	$360 (12)	$180 (6)
			Serum	90^2d^	0.81	NA			$495	NA		
Disease elimination (confirming lack of *M. hyopneumoniae* in resident population)		Two-times test (3 and 1 week prior to the end of herd closure)	Trachea	120^2e^ from the last population of exposed females	0.47	0.46	0.44	0.45	$7200	$2400 (40)	$1440 (48)	$720 (24)

^1^The comparisons of probability of detection estimates are done within a diagnostic testing scenario. Probabilities of detection estimates vary based on sample type, sample size, the time between *M. hyopneumoniae* presence in the population and sampling, and if it is tested individually or pooled.

^2^For the quarantine scenario (2a), time between pigs’ placement and sampling is represented by “@ days (d), e.g., 120 tracheal samples collected at 21 days of placement. For the breeding stock that markets weaned pigs (2b), the probability of detection results from the assumption that *M. hyopneumoniae* has been in the sow population for 21 days. For grow-out sites, the probability of detection results from the assumption that *M. hyopneumoniae* has been in the population for 42 (2c) and 49 days (2d). For disease elimination, the probability of detection results from the assumption that *M. hyopneumoniae* has been in the population for 7 days (i.e., low prevalence scenario).

^3^Probability of detection estimates from individual tracheal testing was extracted from Clavijo et al.^23^ while from pooling tests was extracted from Table 2.

^4^Testing cost was assumed to be $5.50 (United States of American [USA] dollar) for serum tested by ELISA and $30 (USA dollar) per test for tracheal by PCR.

In scenario 2, the aim is to conduct active surveillance on farms specializing in producing replacement pigs, such as multipliers. In farms marketing weaned pigs (continuous flow), a monthly testing of sows is typically performed. If *M. hyopneumoniae* has been present in the sow population for 21 days, collecting 30 or 60 tracheal samples from sows and using a pool size of 10 resulted in 0.39 or 0.61 probability of detection and a cost of $90 or $180, respectively. In contrast, farms that market grow-out pigs (all-in all-out scenario) face a lower risk of sending positive pigs because there is more time for disease detection in all-in all-out growing sites. In this scenario, by extending the time to test samples (e.g., 42 days), collecting 30 or 60 tracheal samples resulted in 0.74 or 0.91, respectively, using a pool size of 5.

## Discussion

Developing an optimal diagnostic protocol hinges on several test-related parameters, such as, sampling objective, sample size, pathogen prevalence, test performance, as well as economic factors such as, labor constraints, risk of false negative/positive results, and sampling and testing cost. In pig production, timely identification of positive replacement pigs (e.g., gilts or boars) is critical in *M. hyopneumoniae*-naïve herds. Accurate and economical surveillance programs are needed to monitor herd status. However, the intrinsic nature of *M. hyopneumoniae*, specifically, its low transmission rate, complicates timely detection during critical periods, such as elimination programs or quarantine testing prior to introduction of replacement pigs^23–25,41,46^. Although tracheal samples tested by PCR are the most sensitive and specific ante-mortem specimen for *M. hyopneumoniae* detection, large sample sizes are needed to have satisfactory probabilities of detection^23,27^. Individual testing of large sample sizes may be financially prohibitive due to the high cost of PCR testing (~ $30) on a per sample basis. To minimize the economic strain of testing a high volume of samples, strategies such as pooling samples or implementing aggregate sample types (i.e., oral fluids), have been effective for detection of other swine diseases, such as PRRSV^45^. However, in the specific case of *M. hyopneumoniae* detection in oral fluids, previous studies have shown a low probability of detection during early to mid-stages of infection, limiting its use in most testing scenarios^23,27,46^.

In this study, three pooling approaches (i.e., pool sizes of 3, 5, and 10) were evaluated to determine their sensitivity in detecting *M. hyopneumoniae* at varying levels of PCR Cts. These data were used to develop novel probability of detection estimates for the three different pooling strategies. Pooling of tracheal samples was shown to be consistent in the detection of *M. hyopneumoniae* DNA in all evaluated pool sizes for individual Ct values of 33 or lower (all but one sample resulted in a negative resulted when the Ct value of individual tracheal sample was 33). If a pool of 3, 5, or 10 had one positive sample with a Ct value of 33 or below, the pool resulted in a positive result, regardless of the pool size. This may be due to the dilution effect of the pool to the original sample and analytical sensitivity of the test^35^. Thus, it is expected that dilution of positive samples can reduce the nucleic acid concentration load below the assay detection limit. However, the results of this study indicate that individual samples with a Ct value of 33 or below are detectable in pools of 3, 5 and 10. Poeta Silva et al.^47^ showed that 4.7% (10/212) tracheal samples from *M. hyopneumoniae*-inoculated pigs resulted in Ct > 33 at the start (3 days post-inoculation) and late (52 days post-inoculation) stage of infection in a controlled setting. Clavijo et al.^23^ reported that 21 of 130 *M. hyopneumoniae* PCR-positive tracheal samples collected from growing pigs naturally exposed to *M. hyopneumoniae* obtained a Ct > 31. Betlach et al.^25^ showed that *M. hyopneumoniae*-inoculated gilts obtained PCR Cts ranging between 20 to 25 on 7 days post-inoculation, while contact gilts obtained PCR Cts 35.7 after 23 days post-contact with seeder gilts. Additionally, data from 286 porcine diagnostic submissions including individual tracheal samples from 2010 to 2021 across four swine-focused Veterinary Diagnostic Laboratories showed that 16.8% obtained average Ct values between 34 and 36 (Swine Disease Reporting System, unpublished data). These data demonstrate that most field samples are commonly tested at a Ct of 33 or below. Given this and the results observed in this study, practitioners may pool up to 10 samples and expect a high probability of detecting *M. hyopneumoniae*. However, these data show that false negative results may occur and should be taken into consideration in high-risk scenarios.

In accordance with Sponheim et al.^42^, the results of this study also supported the use of tracheal samples in pools of 3 and 5. Additionally, this study evaluated the sensitivity of pooling samples by 10, given the high sample sizes required for detection in low prevalence scenarios. Differences in the *M. hyopneumoniae* DNA detection rate were observed between studies, e.g., Sponheim et al.^42^ reported 84% (42 of 50 tracheal samples) detection rate and 0.84 (95% confidence interval 0.71–0.93) pool sensitivity in pools of 3, and 82% (41 of 50 tracheal samples) and 0.82 (95% confidence interval 0.69–0.913) in pools of 5. In contrast, this study found that detection rates and pool sensitivity were higher, 137 of 144 (95.1%) positive tracheal samples in pools of 3, 136 of 144 (94.4%) positive tracheal samples in pools of 5, and 130 of 144 (90.3%) positive tracheal samples in pools of 10. These differences can be attributed to variance in PCR test protocol (i.e., different primers and procedures) and initial PCR Ct values of the individual samples. Sponheim et al.^42^ reported Ct values ranging between ~ 19 through ~ 37 for tracheal samples in pools of 3. A side-by-side comparison of *M. hyopneumoniae* DNA detection in pen-based oral fluids showed that four PCR protocols can vary significantly in detection rate and Ct values^48^. As shown by both studies, false negative results from a pooled sample are possible.

Probability of detection estimates are crucial for the design of feasible, sensitive, and effective surveillance programs centered at verifying freedom of disease in negative populations. These estimates are reflective of the test performance with varying sample types, sample sizes, and prevalence. Clavijo et al.^23^ used simulated field data to show that tracheal samples provided the highest probability of detection estimates for *M. hyopneumoniae*, particularly within the first 21 days of infection, but at a significantly higher cost compared to using serum and oral fluid samples. That is, to detect at least one *M. hyopneumoniae*-infected pig in a 1,250-head wean-to-finishing population, 60 tracheal samples at 28 days post-infection resulted in a probability of detection of 76% (Table 2), with a cost of US$1,800 (assuming $30 per PCR sample). In this study, regardless of pool size, sample size or days post-infection, probability of detection estimates remained relatively similar (i.e., within 5%) compared to the individual estimates previously published (Table 2)^23^. For instance, 30 tracheal samples, a very common sample size utilized by the industry, individually tested at 21 days post-infection (i.e., assuming the agent has been in the population for 21 days) provides a 41% probability of detection, for roughly $900. For the same scenario but utilizing a pooling approach, pools of 3 or 5 provided a probability of detection of 40%, and 39% for pools of 10. These results indicate that a practitioner can collect larger sample sizes and implement a pooling strategy and maintain the same probability of detection as testing individual samples. This approach allows for more animals to be tested without economic testing constraints. However, as seen in Table 3, larger sample sizes are needed to obtain high probability of detection estimates for early detection of the agent (i.e., within 21 days), primarily due to the low transmission rate of *M. hyopneumoniae*^24,25,41^. Implementing a pooling strategy proves effective in reducing testing expenses without compromising the probability of detection. However, the labor cost is not reduced given the necessity for skilled personnel to consistently collect a substantial number of tracheal samples remains indispensable. Resources on proper sample collection are publicly available through the Certified Swine Sample Collector Training Program. This program aims to equip industry stakeholders with the necessary expertise to collect and submit various diagnostic samples during an outbreak, thereby expanding the pool of individuals capable of performing this crucial task and reducing the reliance on veterinarians^49^. By fostering such programs, the capacity of specialized individuals required for *M. hyopneumoniae* surveillance can be significantly enhanced.

Incorporation of external pigs into a production system poses the most significant risk for disease introduction. Therefore, implementing sensitive quarantine testing schemes becomes crucial to safeguard the herd’s health. Considering a 30-day quarantine period, a testing approach involving 120 tracheal swabs at 21 days post-introduction could be employed with an 84% probability of detection (Table 3). Moreover, if adopting a pooling approach, the probability of detection only marginally decreases when pooling samples by 5 or 10, resulting in significantly reduced testing costs. The absence of high probability estimates during the early disease phase might lead swine practitioners to contemplate alternative strategies, such as extending the quarantine duration to reduce sample size or substituting tracheal samples with more convenient and economical options, like serum sampling. For instance, collecting 60 tracheal samples at day 35 post-introduction, pooled by 3, yields an 86% probability of detection, with half of the sample size, labor, and test cost required when compared to collected 120 tracheal samples collected at 21 days post-inoculation (Table 3). This estimate remains the same if the quarantine is extended to around 49 days. Alternatively, if practitioners opt for an alternative sample type, such as serum, testing 120 samples at day 35, 42, or 49 (assuming an extended quarantine) results in probabilities of detection of 60%, 79%, and 88%, respectively.

For multiplication breeding herds, active surveillance programs have traditionally employed a fixed sample size, usually of serum samples, collected monthly (Table 3). The main goal is to identify disease outbreaks within a 30-day timeframe. However, due to the delayed seroconversion and then serum antibody detection of *M. hyopneumoniae* infections, there is a significant possibility of missing an outbreak if it occurred less than 21 days before testing^27,29,30,50^. Hence, choosing an appropriate sample size requires careful consideration of balancing risk and test cost. Herds that frequently market wean age-pigs would benefit from an increased sample size to detect diseases earlier and prevent the shipment of *M. hyopneumoniae*-positive pigs. For instance, if *M. hyopneumoniae* has been present for 21 days, using 30 or 60 tracheal samples pooled by 5 or 10 would yield approximately 40% or 61% probability of detection, respectively. Pooling by 5 or 10 comes with a cost of $360 or $180 per site per month, respectively, with a minimal reduction in the probability of detection compared to individual testing. This limited likelihood of detection (40–60%), reinforces the idea that, for production systems receiving weaned pigs, emphasis should be placed on allocating surveillance resources to the quarantine or during the gilt grow-out period. On the other hand, sow herds that do not market weaned pigs may opt for a higher risk approach, change in sample type, such as serum, involving a lower sample size, and focus their diagnostic resources on grow-out sites from which pigs are marketed (Table 3). This strategy allows them to allocate their resources more efficiently for risk management (Table 3).

*M. hyopneumoniae* elimination programs continue to expand across the industry^20^. Such efforts come with substantial costs, ranging from $67,000 to $112,000, attributed to factors such as loss of sow productivity, medication expenses, and the need for an off-site breeding project^21,51^. Consequently, there is a pressing need for highly sensitive and feasible diagnostic protocols capable of detecting *M. hyopneumoniae* at the culmination of an elimination program^23,27,46^. Timely detection of the agent before introducing naïve replacement females could potentially allow for an extension of the herd closure until shedding has ceased. For instance, the collection of 120 tracheal samples from the last exposed females before herd closure, at 3 and 1 week prior to introducing negative replacement females, can provide an approximately 45% probability of detection per testing, assuming a low prevalence (Table 3). The rather low probability of detection highlights the challenge of *M. hyopneumoniae* detection in very low prevalence scenarios. Thus, a higher sample size or repeated samplings should be considered to increase the probability of detection prior to the introduction of naïve replacements.

## Conclusion

Given the substantial cost linked to *M. hyopneumoniae* infection, the increasing adoption of elimination programs, and the consequent rise in naïve herds, there is a pressing need for precise, practical, and cost-effective surveillance programs. This study showed that pooling sizes of 3, 5 and 10 could be a valuable tool in surveillance for *M. hyopneumoniae*, without significant impact on diagnostic sensitivity or probability of detection estimates, compared to an individual testing strategy while lowering substantially the diagnostic cost. The study also highlighted the increased labor (veterinarian costs) due to the large sample sizes needed for early detection, which can be partially alleviated by training on-farm employees. Furthermore, this manuscript applies the probability of detection estimates on various real-life testing scenarios. However, estimating the potential prevalence of *M. hyopneumoniae* at any given scenario before pooling is crucial for pool size selection. Taken together, surveillance programs can incorporate tracheal pooling as a cost-effect strategy to maximize detection of *M. hyopneumoniae* in negative populations.

## Data Availability

The datasets presented in this article are available upon reasonable request and should be directed to Maria Jose Clavijo, mclavijo@iastate.edu.
